# QUALITY OF WORK LIFE FOR CARE AIDES IN CANADIAN LONG-TERM CARE HOMES BEFORE AND DURING THE PANDEMIC

**DOI:** 10.1093/geroni/igad104.1852

**Published:** 2023-12-21

## Abstract

Canadian Long-term care (LTC) homes were profoundly affected by the COVID-19 pandemic influencing work outcomes of care aides (CAs) who provide most direct care in these homes. We compared CAs’ demographics and quality of work life (QWL) before and during the pandemic by conducting a repeated cross-sectional analysis of data collected in February 2020 (pre-pandemic) and December 2021 (21 months later) from a stratified random sample of urban LTC homes in western Canada. 2348 and 1116 CAs completed the survey in 2020 and 2021, respectively about their work outcomes (e.g., working short, tasks left undone, burnout) and health. We used three-level mixed-effects regression models to compare CAs’ QWL, accounting for repeated-measures and Cas within same care units . Models also adjusted for CA demographics and LTC characteristics. Compared to the 2020 sample, the 2021 sample were 1.56 times more likely to report having worked short-staffed daily-weekly. The 2021 sample of CAs also reported lower levels of professional efficacy and mental health, being less rushed, and experienced fewer responsive behaviors from residents than the 2020 sample. Our prior research has demonstrated stable professional efficacy among CAs over 15 years. In this study, we observed that cracks in CAs’ resolve are starting to show (as supported by their decreased efficacy and mental health). Interventions that address long-standing undervaluing of this staff group are needed as are interventions to support improvement in staff mental health.

